# Developing, Integrating, and Implementing Evidence-Informed Practice Curricula Throughout a Chinese Medicine Degree Program

**DOI:** 10.1089/acm.2019.0456

**Published:** 2020-06-10

**Authors:** Belinda J. Anderson, Benjamin E. Kligler, Paul R. Marantz, Stacy Gomes, William J. Casalaina, Marianne Fuenmayor, Jason Ginsberg, Kathleen A. Greenough, Olga Reznikova, Robert L. Saronson, Helen Zhang, Roni Evans

**Affiliations:** ^1^ Pacific College of Oriental Medicine, New York, NY, USA.; ^2^ Albert Einstein College of Medicine, Bronx, NY, USA.; ^3^ University of Minnesota, Minneapolis, MN, USA.

**Keywords:** Chinese medicine, acupuncture, education, evidence-based medicine, evidence informed practice, faculty training, curriculum development

## Abstract

***Objectives:*** To train faculty and develop curricula in evidence-informed practice (EIP) within a Chinese medicine degree program.

***Setting:*** Pacific College of Oriental Medicine (New York).

***Design:*** Faculty EIP training was undertaken through utilization of online EIP modules, and development and completion of a 3-credit (45 h) online Foundations of EIP course. This was supplemented by faculty meetings and one-on-one support from department chairs. Curriculum development was undertaken by an EIP Curriculum Committee. The committee followed a modified Delphi process to develop EIP course learning outcomes (CLOs), and to make changes to the College's clinic policies and procedures. EIP assignments were developed for each course in accordance with the CLO.

***Results:*** Ninety-one percent of the faculty and 97% of clinical supervisors received formal EIP training. Thirty-five percent of all didactic faculty, 38% of faculty teaching courses with EIP incorporated, and 30% of clinical supervisors completed 10 or more h of EIP training during this project. Faculty also received informal EIP training through participation in department and general faculty meetings. Seventy-three percent of the Master's degree curriculum, inclusive of 40 didactic courses and fifteen 60-h clinic shifts, were modified to incorporate EIP. EIP CLOs and corresponding assignments were developed. Clinic intake forms were modified to facilitate undertaking EIP in the College clinic. Issues related to how EIP is defined in conjunction with the nature of available scientific research in Chinese medicine required discussion and resolution.

***Conclusions:*** Training faculty and developing curricula in EIP within Chinese medicine colleges has unique challenges that must be factored into the strategies and processes. Factors that contributed to the success of this project were having faculty drive the process, integrating EIP content within existing curricula, gradual exposure, identifying champions, relating EIP to practice building, and openly discussing opposing perspectives.

## Introduction

Using evidence to inform clinical practice is an important foundational principle in biomedical education and practice.^1^ Evidence-based medicine (EBM) dictates that treatment approaches are formulated based on a process of critical thinking that utilizes the best available scientific evidence, clinical experience, and an understanding of patient preferences. Complementary and integrative health (CIH) also uses this principle, but the incorporation of scientific evidence is more challenging due to several factors.^2–5^ A lack of research literacy among CIH practitioners^6^ is an important factor that the National Institutes of Health (NIH) addressed in the development of an R25 grant mechanism that was designed to provide funds to complementary and integrative medical colleges to train faculty and develop curricula in research literacy and EBM.^7,8^ Nine institutions received these grants and successfully implemented these educational initiatives within chiropractic, Chinese medicine, massage therapy, naturopathic, and osteopathic degree programs.^8,9^

One such institution was Northwestern Health Sciences University (NWHSU),^10^ which later partnered with Pacific College of Oriental Medicine (PCOM) and Albert Einstein College of Medicine to undertake a similar project at the PCOM campus in New York city. This was supported by a grant from the NIH National Center for Complementary and Integrative Health (NCCIH). Previous similar projects at Oregon College of Oriental Medicine (OCOM)^11^ and the New England School of Acupuncture (NESA)^12^ were successful in engaging students and faculty in research training, but they did also report a certain level of resistance. Previous studies at PCOM had demonstrated that faculty and students also had reservations about research. To directly address this issue and to lessen its potentially deleterious impact on the project, an emphasis was placed on trying to better understand the faculty and student's perspectives surrounding research and EBM. Insights gained from this were then used to incentivize faculty and students, and develop curricula.

A cross-sectional analysis of faculty and students at PCOM-NY had shown that although there was a high degree of interest and support for research and EBM, there were also concerns about the relevance of research to Chinese medicine, and the possibility of cooptation by biomedicine.^13,14^ Among the students, their interest and support for research declined as they progressed through the 4 years of training.^14^ A similar decline has also been shown at OCOM and NESA.^15^ A later qualitative analysis of PCOM alumni, who were experienced acupuncturists undertaking a doctoral degree at PCOM, also showed concerns around research and EBM.^16^ Similar to the other studies, these concerns centered around the relevance of research to clinical practice and cooptation, but they also focused on paradigm differences, the validity of clinical trial methodology, and power imbalances in the health care arena.^2–5^

The Chinese medicine programs at PCOM contain research courses, which is not an accreditation requirement at the Master's degree level. PCOM's programs also have a greater number of biomedical credits than is required to meet accreditation standards. This reflects PCOM's focus on integrative medicine, and the recognition that acupuncturists require a high level of biomedical competency. Our studies^13,14^ found that >70% of both the faculty and fourth-year students agreed, or strongly agreed, that they knew how to find and critique the research literature, and more than 58% of the faculty reported to be reading journal articles every week. A faculty needs assessment, similar to that undertaken at NWHSU,^10^ was undertaken in the first few months of the project. This also showed high levels of faculty self-reported research literacy, and support for the importance of research. However, faculty again expressed concerns around paradigm differences, and the ability to integrate research into the discipline.

The purpose of the project was to build on the basic research competency of the faculty and students, and focus especially on the use and application of research findings to clinical practice. This article outlines how faculty research and EBM training were undertaken at PCOM-NY, and how the curriculum of the Master's degree in Chinese medicine was modified to incorporate EBM principles in both a stand-alone course and embedded throughout the didactic and clinical curricula. This article uses the term “evidence informed practice” (EIP) along with EBM, because EIP was the term adopted by NWHSU.

## Materials and Methods

### Development of the Foundations of EIP course

Two 45-h, 3-credit Foundations of EIP courses were developed by the principal investigator (PI; BA) for either in-person or online synchronous instruction. These courses had the same course learning outcomes (CLOs), content, and similar assignments. Both courses were based on a similar course that was developed by NWHSU in collaboration with the University of Minnesota (UMN) under their NIH NCCIH R25 grant.^10,17^ Syllabi, slides, and course assignments from this course were used to create the Foundations of EIP courses. Modifications were made to enhance direct relevance to Chinese medicine, and for the online course, to facilitate online instruction. Modification to enhance relevance to Chinese medicine mainly consisted of using acupuncture therapy and Chinese herbal medicine research to illustrate concepts, and for inclusion in assessment instruments. Both Foundations of EIP courses also incorporated the EIP online educational materials (EIP modules)^17^ that were developed through the R25 grant, which are now housed at the UMN and consist of 10 modules that take ∼10 h to complete.^18^ The online EIP modules contain test questions throughout and at the end of each module, enabling self-testing of knowledge. Use was also made of the CARE guidelines,^19^ which were developed to make reporting of case studies more accurate, transparent, and useful.

### Faculty EIP training

The basis of the faculty EIP training approach was the use of the online EIP modules, EIP in-service training in faculty meetings, mentoring from department chairs, and voluntary participation in a journal club. Faculty completed either all the modules (∼10 h of EIP instruction) or our required introductory minimum training in EIP (4 modules, 1.3 h of EIP instruction). Some elected to complete the full, 45-h Foundations of EIP course.

All faculty teaching courses with EIP content, and all clinical supervisors, were asked to undertake EIP training. Faculty were paid by the College for the time that it took to undertake the EIP training (inclusive of the modules, in-service training, and one-on-one meetings) and received continuing education credit for EIP module completion from the National Certification Commission for Acupuncture and Oriental Medicine. Some faculty undertook PCOM's online doctoral degree, which was designed to enable Master's-qualified graduates to upgrade to a doctoral degree. This degree included the 45-h, 3-credit, online Foundations of EIP course.

The project's PI (also the Academic Dean at PCOM-NY) presented EIP trainings in all department and general faculty meetings. The one-on-one faculty EIP training was undertaken by the department chairs, who were required to have completed all 10 EIP modules and were members of the EIP Curriculum Committee. The department chairs trained faculty by face-to-face meetings, class visits, and e-mail communication.

### EIP Curriculum Development Committee

An EIP Curriculum Committee was formed 2.5 years into the 5-year grant. The committee consisted of the PI, all department chairs, chair of faculty governance, two full-time faculty, and one adjunct faculty. Committee meetings were for 1 h and the committee met twice in year 3, five times in year 4, and six times in year 5.

#### EIP curriculum mapping

The Chinese medicine program that was the focus of this project is called the Master of Science in Traditional Oriental Medicine (MSTOM), and it includes curricula in biomedicine, acupuncture therapy, and Chinese herbal medicine. The committee first assessed the didactic and clinical MSTOM program learning outcomes, and was satisfied that sufficient EIP content was represented. The program's individual courses were then examined to determine which courses would be the best candidates for EIP inclusion.

#### EIP CLO development

Department chairs were tasked with creating CLOs for these courses. This process was initiated by the PI, who created a list of the types of EIP activities that could be included in these courses, and their corresponding CLOs. These CLOs were based on EBM competencies that recipients of the prior R25 NIH grants had established were the most important.^9^ These included articulating answerable clinical questions, gathering evidence from the research and other appropriate sources, critically evaluating the evidence, and interpreting and applying it within a clinical context.

Department chairs collaborated with the PI and other members of the EIP Curriculum Committee in the creation of the CLOs. On completion, the CLOs were further discussed and modified during committee meetings. Modifications consisted of minor changes to wording and more significant changes such as using the word “literature” instead of “research literature,” so Chinese medicine sources were equally as applicable as research studies. This first draft of the EIP CLOs was then shared with PCOM's two other campuses (in San Diego and Chicago), who assessed them and provided feedback. Several rounds of consensus building using a modified Delphi process^20^ among the campuses were undertaken to achieve consensus on the final set of EIP CLOs.

### Modification of clinic policies and procedures to incorporate EIP

The Accreditation Commission for Acupuncture and Oriental Medicine requires colleges offering programs in Chinese medicine to have clinics in which students and faculty provide Chinese medicine treatments to fee-paying patients. To meet accreditation requirements, students in the MSTOM are required to perform a minimum of 350 treatments on a minimum of 50 different patients throughout the degree program.

Discussions about how to incorporate EIP into the clinic occurred between the PI, the director of the clinic, and the clinical department chair during several meetings in the 4th and 5th years of the 5-year grant. These meetings centered around modification of patient intake forms, provision of resources in the clinic to facilitate EIP, and adoption of clinical outcome instruments.

### Journal club

The PI identified a full-time faculty member who had a high level of enthusiasm for EIP to establish and manage a journal club. The purpose of the club was to provide additional opportunities for faculty and students to engage with research, and to raise EIP awareness on campus.

## Results

### Foundations of EIP course

The CLOs and assignments that were developed for the Foundations of EIP course are shown in Table 1. The course was designed to train students to be informed and astute consumers of research. It was not designed to provide basic instruction in how to conduct research. The importance of the use of relevant evidence to inform clinical practice was emphasized, along with combining research evidence with patient preferences and clinician experience. Finding research, assessing its relevance and validity, and applying it to clinical practice were topics that were covered in detail by using studies that focused on acupuncture. Assessing relevance also included analysis and comparison of different clinical trial methodologies, and their appropriateness to acupuncture practice within real-world settings. The use of research evidence to build a Chinese medicine practice through marketing, professional relationship-building, and presentations was utilized to demonstrate the broad value of EIP.

**Table 1.** Foundations of Evidence-Informed Practice Course

CLOs	(1) Describe fundamental principles of research.	
	(2) Define EIP and discuss its importance in health care delivery.	
	(3) Find and appraise different forms of evidence.	
	(4) Discuss the importance of research in one's discipline and its current status.	
	(5) Apply relevant research evidence, patient preferences, and clinical experience in clinical practice.	
		*% Final grade*
Assignments	Discussion of two published EIP opinion articles through online forums	15
	Finding and summarizing research articles	9
	Using a validated outcome instrument in students' clinical work for patient evaluation	10
	Describing the application of EIP to clinical practice or teaching	10
	Class debate—for and against EIP	10
	Case study using the CARE^a^ case report guidelines	15
	Seven in-class small group exercises finding and evaluating different evidence sources	21
	Final exam	10

^a^ www.care-statement.org

CLO, course learning outcome; EIP, evidence informed practice.

The course used several modes of instruction: lecture, independent study, group work, and student presentations. Polls to canvas responses to multiple choice and open-ended questions were also used in the online course to make it more interactive. The assessment consisted of discussion of published EIP opinion articles, finding and assessing research articles, utilizing and assessing outcome instruments, creating a plan for applying EIP clinically or for teaching purposes (in the online course that had licensed acupuncturists and faculty as students), a class debate for and against EIP, writing up a case study using the CARE guidelines,^19^ and a short exam on the material covered in the EIP modules.

### Faculty training outcomes

Table 2 presents the outcomes of the formal faculty EIP training initiative. Of the 70 faculty, 60 were teaching courses that were identified for EIP incorporation, and 37 were clinical supervisors. Of the didactic (classroom) faculty, 91% of the total faculty and 97% of the faculty teaching courses with EIP content undertook the required EIP training (a minimum of four EIP modules), or had previously completed EIP training as part of a relevant doctoral degree. In both groups, almost half exceeded the minimum training by completing 10 or more h of EIP training, of which 31% of the total faculty and 35% of the faculty teaching courses with EIP content completed the 3-credit (45-h) Foundations of EIP course. Ninety-seven percent of the clinical faculty completed EIP training, with 30% completing the 3-credit (45-h) Foundations of EIP course.

**Table 2.** Pacific College of Oriental Medicine-New York Faculty Evidence-Informed Practice Training Outcomes

EIP training access	% of PCOM-NY Chinese medicine faculty		
	Didactic (classroom) faculty		Clinical supervisors
	% of Total (70)	% of Required (60)^a^	% of clinical supervisors (37)
FEIP 3-CR course (42 h)	31% (22)	35% (21)	30% (11)
All EIP modules (10 h)	4% (3)	3% (2)	0% (0)
Four EIP modules (1.3 h)	50% (35)	55% (32)	65% (24)
Exempt—doctoral degree	6% (4)	5% (3)	3% (1)
Total	91% (64)	97% (58)	97% (36)

^a^ 60 of the 70 faculty were teaching courses that were selected for EIP incorporation.

CR, credit; PCOM, Pacific College of Oriental Medicine.

### EIP curriculum mapping, CLOs, and assignments

Table 3 presents the didactic and clinical courses that were identified by the EIP Curriculum Committee for inclusion of EIP content and corresponding EIP CLOs. This includes 40 of the 58 didactic courses and all (15) of the 60-h clinic shifts. Overall, EIP was introduced into 130.25 of the 178.5 credits in the program, which represents 73% of the MSTOM degree curriculum. All biomedical courses except some of the anatomy and physiology courses, and all clinical practice courses were modified to include EIP. In the remaining non-biomedical disciplines, the majority of the courses were also modified to include EIP. Courses that were deemed unsuitable for EIP inclusion were mainly those that covered foundational, discipline-specific material in the early stages of the degree program, and occurred before basic EIP training was provided.

**Table 3.** Curriculum Mapping—Identification of Courses for Evidence-Informed Practice Inclusion and Defining Course Learning Outcomes

Course name and number of credits (CR)	EIP CLOs
***Biomedicine***	
Medical Terminology (1.5 CR)	Understand how to find relevant research literature
Biology (2.5 CR)	(1) Understand how to find relevant research literature
	(2) Analyze basic science research findings and determine how they relate to the mechanisms of normal and altered states of health
Survey of Biochemical Principles (2 CR)	Demonstrate the ability to find, analyze, and summarize relevant research evidence regarding biochemical mechanisms of Chinese medicine
Anatomy and Physiology 2 (3 CR)	Analyze basic science research findings according to how they apply to the mechanisms of acupuncture and other Chinese medicine modalities
Biological Aspects of Physics (2.5 CR)	
Eastern & Western Nutrition (3 CR)	Devise a nutritional treatment plan and outcome assessment by using case-relevant research
Pathophysiology 1, 2 (3 CR each)	Apply best practice clinical research literature to support diagnosis and treatment decisions
Ortho-Neuro Evaluation 1, 2 (2 CR each)	
Pharmacology (2 CR)	
Clinical Science (3 CR)	
Physical Exam (2.5 CR)	Apply best practice literature to support diagnosis and treatment decisions
Medical-Legal Report Writing (2 CR)	Incorporate EIP in communication of medical-legal reports with patients, other health care providers, and third-party payers
***Acupuncture***	
Acupuncture. Channels & Points 4—Auricular (0.75 CR)	Find analyze, and summarize literature relevant to the scientific and classical understanding of auricular acupuncture
Introduction to Clinical Techniques (2.5 CR)	Find, analyze, and summarize literature relevant to the clinical techniques used in Chinese medicine
Needle Technique (2.5 CR)	Find, analyze, and summarize literature relevant to the understanding of acupuncture and adjunctive techniques
Advanced Needle Techniques (2.5 CR)	Find analyze, and summarize literature relevant to the understanding of classical and modern acupuncture techniques, including e-stim and scalp acupuncture
Treatment of Orthopedic Disorders (2 CR)	Find, analyze, and summarize clinical findings of literature relevant to treating orthopedic disorders with acupuncture and other Chinese medicine modalities
***Oriental Medicine***	
Philosophical & Historical Foundations of Chinese Medicine (3 CR)	Differentiate primary from secondary, and modern from premodern sources for the study of traditional East Asian medicine.
Diagnosis & Treatment of Disease 1-5 (3 CR each)	Devise a treatment plan and outcome assessment incorporating case relevant research
***Herbal Medicine***	
Introduction to Herbology (2 CR)	(1) Understand how to find relevant literature
	(2) Understand best practice literature and how it applies to the practice of Chinese herbal medicine
Herbology 4-6 (3 CR each)	Apply best practice literature to support diagnosis and treatment decisions
Chinese Herbs & Internal Med. 1-3 (3 CR each)	Devise a treatment plan and outcome assessment by using case-relevant best practice literature
***Movement and Manual Therapy Courses***	
Tai Ji/Qigong (1.5 CR)	Perform a search of scientific databases and find relevant literature
Tui Na Hand & Structural Techniques (1.5 CR each)	
***Clinical Practice***	
Clinical Counseling 2 (1.5 CR)	Find, analyze, and summarize relevant clinical research literature findings
Practice Management & Ethics (3 CR)	Demonstrate the use of EIP for marketing and patient retention, inclusive of communication with patients, other health care providers, third-party payers, and the public
Introduction to Clinical Observation (1 CR)	Describe/explain the tools and resources of EIP
Introduction to Associate Internship (1 CR)	Devise treatment and assessment recommendations using the basic principles of EIP—Analyze, Ask (PICO), Acquire, Appraise, Apply, Assess
Associate Internship Grand Rounds (1 CR)	
Clinical Observation (2 CR)	Perform a search of scientific databases and find relevant research literature

Department chairs, in collaboration with the PI and faculty, created the first draft of the CLOs for all the courses and clinic shifts. These were discussed and modified throughout a 6-month period inclusive of four meetings of the EIP Curriculum Committee. The committee discussions focused around what EIP content was appropriate for each course according to its discipline and chronological place in the program curriculum, and the importance of inclusiveness with regard to all evidence sources. This latter issue was an especially sensitive topic, because Chinese medicine is thousands of years old with a vast body of clinical instruction literature. The committee wanted the CLOs to reflect that evidence could come from modern scientific and/or from Chinese medicine sources. The CLOs emphasized the importance of critical thinking—engaging in an evidence-based reasoning process to substantiate clinical decisions.

When the NY campus EIP Curriculum Committee had completed its edits to the CLOs, they were presented to the San Diego and Chicago campus deans who shared them with their faculty, and later sent feedback to the Committee. Three rounds of back-and-forth discussion and editing took place between the Committee and the San Diego campus, and one round with the Chicago campus over a 6-month period. The final CLOs for the 40 didactic courses are shown in Table 3.

After agreement on the EIP CLOs, the NY campus department chairs worked with faculty to develop EIP assignments. Table 4 presents examples of the EIP assignments that were added to these courses, along with the percentage of the final grade each was worth, which ranged from 2% to 20%. Some assignments were embedded within larger assignments (e.g., within a case study or an exam), and some were stand-alone EIP assignments.

**Table 4.** Description of Evidence-Informed Practice Assignments and Percentage of the Final Course Grade

Course name and number of credits (CR)	EIP assignment	% Final grade
*Biomedicine*		
Medical Terminology (1.5 CR)	Identify National Library of Medicine Medical Subject Headings (MeSH) in scientific publications	2
Biology (2.5 CR)	Search for scientific information and research literature on various aspects of the topic of malaria	10
Anatomy and Physiology 2 (3 CR)	Find and summarize a study investigating the neurological mechanism of acupuncture	10
Survey of Biochemical Principles (2 CR)	Assess contraindications of Chinese herbs with specific medical conditions by using relevant scientific studies	5
Biological Aspects of Physics (2.5 CR)	Write a paper outlining the theories for the scientific basis of acupuncture	20
Pathophysiology 1, 2 (3 CR each)	Find and analyze best practice research literature in the context of biomedical diseases and the use of complementary therapies	10
Ortho-Neuro Evaluation 1, 2 (2 CR each)	Propose and support an orthoneurological diagnosis using best practice and research literature	10
Pharmacology (2 CR)	Summarize clinically relevant findings from drug-herb interaction literature	5
Eastern & Western Nutrition (3 CR)	Find and evaluate an original or summary research article related to diet in the “take-out culture” environment	5
Clinical Science (3 CR)	Outline best practice treatment approaches for specific medical conditions	10
Physical Exam (2.5 CR)	Outline best practice approaches to using physical examination techniques in diagnosing abdominal pain	5
Medical-Legal Report Writing (2 CR)	Present a medical report that incorporates evidence to support a referral to a practitioner of a specific medical specialty	10
*Acupuncture*		
Acupuncture Channels & Points 4—Auricular (0.75 CR)	Use Pubmed to find, analyze, and summarize an article about auricular acupuncture therapies	10
Introduction to Clinical Techniques (2.5 CR)	Use Pubmed with provided search terms to find, analyze, and summarize studies relevant to Chinese medicine clinical techniques	5
Needle Technique (2.5 CR)	Use PubMed to find, analyze, and summarize articles about acupuncture and adjunctive therapies	10
Advanced Needle Techniques (2.5 CR)	Use PubMed to find, analyze, and summarize articles assessing the use of e-stim or scalp acupuncture as adjunctive therapies	5
Treatment of Orthopedic Disorders (2 CR)	Use PubMed to find, analyze, and summarize two articles assessing acupuncture and other Chinese medicine modalities for pain management and injury recovery	5
*Oriental Medicine*		
Philosophical and Historical Foundations of Chinese Medicine	Research a cultural, historical, or philosophical topic inclusive of differentiating between primary and secondary, and modern and premodern sources	10
Diagnosis & Treatment of Disease 1-5 (3 CR each)	Devise a treatment plan and outcome assessment incorporating the research literature for a given case study	5
*Herbal Medicine*		
Introduction to Herbology (2 CR)	Knowledge and understanding of research into herbal pharmacology and usage is assessed in quizzes and exams	5
Herbology 4-6 (3 CR each)	Explain the use of different Chinese herbs in the recommended treatment of specific biomedical conditions from a Chinese medicine theoretical perspective	5
Chinese Herbs & Internal Med. 1-3 (3 CR each)	Discuss etiology and pathology of a specific condition by using either a modern or classical Chinese medicine source	4
*Specialty Courses*		
Tai Ji/*Qigong* (1.5 CR)	Write a paper on Tai Ji or *Qigong* including at least 3 references of research studies	10
Tui Na Hand & Structural Techniques (1.5 CR each)	Research an orthopedic condition for a class presentation including recommended treatment and rehabilitation options	8
*Clinical Practice*		
Clinical Counseling 2 (1.5 CR)	Find relevant research literature, and analyze and summarize clinically relevant aspects of topics discussed in the course	5
Practice Management and Ethics (3 CR)	Write a proposal paper for developing a private practice focusing on the use of EIP for marketing, patient retention, building referral networks, and insurance billing	5
Introduction to Clinical Observation (1 CR)	Write a paper defining the concept of EIP including a description of how and where relevant literature would be found	3
Introduction to Associate Internship (1 CR)	Use the basic principles of EIP—analyze, ask (PICO), acquire, appraise, apply, and assess to analyze and write up a patient case	5
Associate Internship Grand Rounds (1 CR)		

PICO, patient problem, intervention, comparison, outcome.

### EIP in the clinic

Table 5 outlines the EIP-related changes that were made to the clinic-based education. Two EIP CLOs were developed. Additions to the clinic intake forms included two additional EIP related questions, and a patient-generated outcome instrument (measure yourself medical outcome profile [MYMOP]).^21,22^ Electronic resources were provided in the clinic consultation rooms to allow faculty and students easy access to evidence sources. Additional EIP competencies were added to the final clinical assessment instrument that is used by clinical supervisors in devising clinic grades.

**Table 5.** Evidence-Informed Practice in the Clinic

Clinic shift CLOs
(1) Apply relevant research evidence in conjunction with patient preferences and clinical expertise in the practice of Chinese medicine.
(2) Demonstrate the use of outcome instruments.
Additions to Clinic intake forms
(1) What evidence and/or clinical experience supports your treatment strategy?
(2) How did patient preferences influence your treatment decisions?
Inclusion of an outcome instrument
MYMOP
Additional resources in the clinic consultation rooms
iPads with ebooks and Apps
Sections of the final clinical assessment that include EIP
(1) Patient care
(2) Medical knowledge
(3) Practice-based learning and improvement

MYMOP, measure yourself medical outcome profile.

### Journal club

The journal club met eight times throughout the last 2.5 years of the grant. The meetings consisted of discussion of research studies and presentations of research by faculty and guest speakers. The average attendance was ∼15 people, with usually more students than faculty. This represented a relatively insignificant aspect of the overall EIP project.

## Discussion

This article describes the success of a Chinese medicine college in engaging more than 90% of its faculty in EBM training, and embedding EBM content into 73% of the Master's degree curriculum. Implementing such faculty training and curriculum changes is challenging in higher education due to faculty resistance and the often-lengthy process required to discuss why change is necessary, and the best way to go about it.^23^ This level of change is, therefore, quite significant. The percentage of the program curriculum where EBM was incorporated was comparable to R25 grant recipients who also infused EBM into more than 60% of their curriculum.^9^ Faculty participation levels in EIP training were similar to those achieved at NWHSU.^10^ Aspects that were critical to the success of this project were engaging and listening to stakeholders' opinions and perspectives and using them strategically, asking faculty to take a lead role in development and implementation, and allowing the profession to integrate EBM within a context that honored the traditions of Chinese medicine.

One of the primary challenges of this project was that it touched on a sensitive issue that is common to colleges that teach CIH; that is, the paradigm differences that exist between Chinese medicine and biomedicine, and the at-times antagonistic and tension-filled relationship between the two.^16,24^ This tension has eased in the past decade, but embracing EBM requires a culture shift within CIH institutions.^25^ Our previous studies demonstrated that faculty and students recognized the importance of research, but they still had concerns related to the relevance of the research to clinical practice.^13,14^ This was also reported at OCOM and the NESA.^15^

The validity of such perspectives is underscored by the fact that much of the published acupuncture research has used placebo-controlled randomized trial methodology. Such trials do not capture the complexity of the real-world practice of Chinese medicine. These trials lack Chinese medicine differential diagnosis using the theories of Chinese medicine, often only use acupuncture and no other modalities that are commonly used in Chinese medicine (such as cupping, moxibustion, Gua Sha, Tui Na etc.), do not individualize treatments, and do not change treatments over time. Consequently, such trials do not provide a valid treatment approach that acupuncturists can relate to within the context of their training.^24,26–28^ Such issues were especially problematic to students and faculty.

For these reasons, the Foundations of EIP course needed to have content that directly addressed this issue. This content included an outline of the history and methodology of scientific research as it has been applied to the field of Chinese medicine. Topics covered included the purpose and weaknesses of the placebo-controlled trial methodology,^27–29^ the development of pragmatic clinical trial models,^29^ comparative effectiveness methodology, whole systems research,^30^ mixed methods, and the use of outcome measures beyond those focused on the condition under investigation, such as quality-of-life measures. A similar approach was used to develop a research course for students at a Canadian Chinese medicine college.^31^ The course included writing a case study using standardized (CARE)^19^ guidelines, which imparted skills for students to engage in their own research. Case writing was also utilized as part of faculty research training at NESA.^11^ Another important component of the Foundations of EIP course that helped to better engage students was discussion about ways in which the research could be used for practice building. This included incorporating research evidence into presentations, and sharing research findings with other health care practitioners and patients.

Faculty engagement, and tasking faculty with taking the lead in developing and implementing projects, is an established strategy in higher education.^32^ Having faculty take such a role in this project was an important strategy in engendering the necessary cultural changes, and it had been used by prior institutions that had received the NIH R25 funding.^8^ Key faculty—the department chairs, chair of faculty governance, and full-time faculty—were required to complete a minimum of all 10 modules of the online EIP training. These faculty then formed the core of the Curriculum Advisory Committee. All faculty were paid for training time and given free continuing educations credits.

Two important facets of this project that facilitated institutional cultural change were the gradual nature of EIP exposure, and openly dealing with, and discussing issues that faculty and students had. Being a 5-year project, faculty were gradually introduced to the concept of EIP and associated curriculum changes. EIP was regularly discussed in all department and faculty meetings well before the faculty were asked to undertake the training or modify their didactic or clinical teaching. This provided time for faculty to express their opinions and concerns. It also allowed time for their perspectives to be incorporated into the way the curriculum was developed and implemented, thereby facilitating their support for the success of the endeavor.

One particular concern of the faculty was the definition of evidence. There was significant resistance to evidence only being defined as that generated by modern scientific studies. Chinese medicine is a several thousand-year-old practice that has been carefully documented. Text books represent the collective clinical experience of thousands of clinicians, and their detailed commentary. Faculty felt this constituted a form of observational research that should be regarded as legitimate evidence. From this perspective, EIP was more appropriately seen as a process of critical thinking. Faculty perceived the curriculum change to be an increased focus on justification for treatment approaches using all available evidence sources. A similar conclusion was reached at OCOM^12^—“In our broader view, research is considered within a spectrum of ‘ways of knowing,’ which includes medical texts (both Oriental medicine and bio-medicine), clinical experience of the practitioner and health care colleagues, patient values, and intuition.”

Additional factors that supported the success of the project were the doctoral-level educational programs that were being developed and began being offered during this project, and the College seeking regional accreditation. Accreditation for the doctoral programs required the inclusion of EBM. This project was a factor in the College, successfully achieving regional accreditation, which allowed the College to offer financial aid for the doctoral programs. These factors highlighted the importance of the project, and they generated increased support from the senior administration. The transitional doctoral program, designed to permit Master's-qualified acupuncturists to upgrade their qualifications to the doctoral level, also lent significant support to faculty EIP training. This was because the College offered a tuition discount for the program to its faculty, many of whom took the program that required them to complete the three-credit Foundations of EIP course. This led to 35% of the faculty teaching courses that had EIP incorporated having taken the Foundations of EIP course, and thereby receiving extensive EBM training. The project PI, who was also the academic dean of the NY campus, taught the Foundations of EIP course, and this thereby provided numerous opportunities for open discussion with faculty about the project.

The main focus of this project was training faculty and developing a didactic EIP curriculum. Assessment of changes in student's EIP skills, knowledge, attitudes, and behaviors before and after taking the Foundations of EIP course, and changes in faculty clinical instruction, were also undertaken and will be reported in a separate forthcoming publication. Translation into the clinic curriculum was started in the last year of the grant and will require ongoing time and focus to support this phase of EIP training. Changing clinical behavior is an especially challenging aspect of EIP training. Many studies have reported poor uptake of EBM in clinical activities after successful EBM didactic training for both biomedical and CIH practitioners.^4,33–35^ This indicates that special focus, and perhaps different strategic methods may be needed for this aspect of EBM training.

There were also important lessons learned and limitations associated this project. Even though all members of the EIP Curriculum Committee had completed the EIP modules, further guidance and discussion around EIP within the context of PCOM's curriculum was critical in enabling them to develop the EIP CLOs and train faculty. The PI's role as dean at the College and an instructor for the Foundations of EIP course permitted ongoing informal evaluation of the faculty experience, which was used in devising strategies to engage faculty and develop curriculum. However, a formal formative evaluation would have also been beneficial.

It was definitely more challenging to get faculty who were very part-time to take the EIP training. Many of these were clinical supervisors, which resulted in a significant proportion of the supervisors (65%) only taking the minimum EIP training (four modules). This is a significant limitation of the project, and it will likely be a barrier to increasing the practice of EIP in the College clinic if further EIP training of clinical supervisors is not undertaken. This project has established an EIP precedence, and increased EIP knowledge and awareness, but long-term sustainability will require ongoing focus in the form of continued faculty training, and further refinement of the didactic EIP curriculum.

## Conclusions

Based on the outcomes of this study, we recommend the following:

(1) Integrating EIP throughout the curriculum rather than creating several stand-alone courses. This reduces accreditation issues and administrative burden, and it creates repetition, consolidation, and gradual application of knowledge and skills.
(2) Engagement of faculty in creating the strategy for undertaking the project.
(3) Gradual engagement of the community, actively seeking out their opinions, and designing the project implementation strategy based on their perspectives.
(4) Identifying faculty champions—people who are supportive and influential who can generate support within the community.
(5) Talking about EBM within the context of practice building and other practical aspects in addition to the clinical applications.
(6) Seriously and authentically embracing negative opinions and making genuine efforts to understand opposing perspectives.
